# Research on Fire Detection of Cotton Picker Based on Improved Algorithm

**DOI:** 10.3390/s25020564

**Published:** 2025-01-19

**Authors:** Zhai Shi, Fangwei Wu, Changjie Han, Dongdong Song

**Affiliations:** College of Mechanical and Electrical Engineering, Xinjiang Agricultural University, Urumqi 830052, China; shizhai-001@126.com (Z.S.); 15715561551@163.com (F.W.); 18293804371@163.com (D.S.)

**Keywords:** fusion algorithm, neural network, cotton pickers, fire detection system

## Abstract

According to the physical characteristics of cotton and the work characteristics of cotton pickers in the field, during the picking process, there is a risk of cotton combustion. The cotton picker working environment is complex, cotton ignition can be hidden, and fire is difficult to detect. Therefore, in this study, we designed an improved algorithm for multi-sensor data fusion; built a cotton picker fire detection system by using infrared temperature sensors, CO sensors, and the upper computer; and proposed a BP neural network model based on improved mutation operator hybrid gray wolf optimizer and particle swarm optimization (MGWO-PSO) algorithm based on the BP neural network model. This algorithm includes the introduction of a mutation operator in the gray wolf algorithm to improve the search ability of the algorithm, and, at the same time, we introduce the PSO algorithm idea. The improved fusion algorithm is used as a learning algorithm to optimize the BP neural network, and the optimized network is used to process and predict the data collected from temperature and gas sensors, which effectively improves the accuracy of fire prediction. The sensor measurements were compared with the actual values to verify the effectiveness of the GWO-PSO-optimized BP neural network model. Once experimentally verified, the improved GWO-PSO algorithm achieves a correlation coefficient R of 0.96929, a prediction accuracy rate of 96.10%, and a prediction error rate of only 3.9%, while the system monitors an accurate early warning rate of 96.07%, and the false alarm and omission rates are both less than 5%. This study can detect cotton picker fires in real time and provide timely warnings, which provides a new method for the accurate detection of fires during the field operation of cotton pickers.

## 1. Introduction

Xinjiang is the main cotton producing area in China. In 2023, Xinjiang’s cotton planting area was 35.54 million mu, with a cotton output of 5.112 million tons, accounting for more than 90% of the country’s total output. In order to improve cotton harvesting efficiency and reduce labor costs, the use of cotton pickers in Xinjiang is increasing year by year, and the demand for cotton pickers is increasing. Machine picking is the main cotton picking method. By 2023, nearly 7000 cotton pickers have been used in Xinjiang to carry out mechanized harvesting [1]. Xinjiang is China’s largest cotton planting area, and the climate is dry. During the picking process, when using cotton pickers, given the friction heat and heat conduction phenomena occurring between the cotton [2], cotton stalks, straw lifters, picking spindles, and doffer grinders, along with the rise in temperature, there is a risk of fire when using cotton pickers.

Currently, research on fire detection for cotton pickers is basically centered around the cotton pipelines and cotton boxes. For instance, Yu [3] considers the working characteristics of cotton pickers and the properties of cotton. The use of infrared detection technology, installed in the cotton pipeline of a cotton picker to detect cotton fires, per Li [4], is crucial in order to realize the early recognition of cotton harvesting operation fires. A mid-infrared carbon monoxide (CO) sensor system was also developed and experimented in the cotton box of a cotton picker. Zhang [5] used a genetic algorithm to optimize the BP network to achieve the detection of cotton picker fire using a sensor installed in the cotton box.

Cotton pickers’ working environments include operational dust, cotton lint, cotton leaves, and other impacts, which affect the use of smoke sensors to collect data from the environment. In the experimental process of smoke collection, data will be affected by dust, other impacts, and false alarms. Thus, using temperature and CO concentration as factors to assess cotton picker fire risk is more effective. According to Shi [6], who analyzed reports of fires in the picking room of a cotton picker, the use of multi-sensors to detect a fire on the cotton picker is of practical significance.

In recent years, machine learning has emerged as a popular area of research exploring how to continuously learn and improve computer systems through experience [7]. For the detection of fire utilizing the data collected by multi-sensor technology, a neural network and machine learning are used to predict fires from the collected data. Neural networks have excellent abilities to learn data features and boast powerful nonlinear mapping abilities and learning abilities [8]. Neural networks have slow convergence speeds and easily fall into the local optimal solution problem; thus, further optimization and improvement of the network are necessary. The literature [9,10,11,12] improves the particle swarm algorithm to optimize the neural network to achieve early fire warnings. Some studies [13,14] optimize the neural network by using the improved gray wolf garlic algorithm. Zhang [5] optimized the BP network by using a genetic algorithm to improve the accuracy of the detection of fires in cotton pickers.

From the current research, there is a paucity of studies on the detection of early fires during the stages of cotton picker operation. Notably, there has been no research specifically targeting the detection of fires resulting from spindle friction at the front end of the picking room of the cotton pickers. In response to this research gap, this paper proposes the installation of sensors in the picking room to detect fire information due to spindle friction in order to more effectively detect fires in the cotton transportation link. In other words, we propose installing infrared temperature and CO sensors near the cotton pipe and cotton box in order to effectively detect fires. In terms of software, the gray wolf–particle swarm optimization BP neural network algorithm with the introduction of variational operators is used for the training of the model through the data collected by the sensors, and the improved algorithm is used to accelerate the convergence of the BP neural network weights and thresholds to obtain the optimal network parameters. The network parameters are derived and then applied to the upper computer of the BP neural network of the cotton pickers to determine fire conditions. The data collected by the sensors, the monitored data, and the fire prediction data are transmitted to the vehicle control terminal system to realize automatic alarms and real-time detection. Before the beginning of the study, the data acquisition and transmission terminal system and the cotton picker fire detection and warning system were designed to lay the experimental foundation for this improved algorithm.

## 2. Materials and Methods

### 2.1. Overall Design of the System

**Figure 1:**
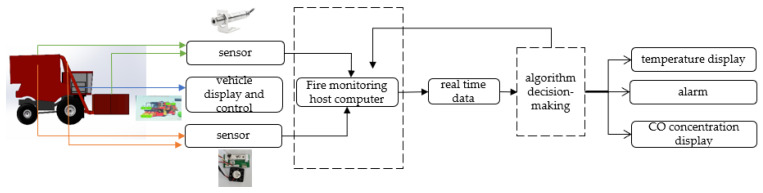
Overall system design diagram.

The cotton picker fire detection system consists of an infrared temperature sensor, a carbon monoxide sensor, an upper computer, and a display screen. According to the analysis of Shi [6] and Qiao [15], it is indicated that during the picking process, the picking spindle generates a significant amount of heat due to high-speed rotation and friction in the winding process, accompanied by an increase in temperature and the generation of CO. The IR sensor and the CO sensor are primarily employed to detect the picking spindle device of cotton pickers. Given that cotton is harvested in the cotton picker through a series of processes involving the picking spindle, the delivery pipe, and the cotton box, the two kinds of sensors are also placed in the delivery pipe and cotton box. The data collected by the multiple sensors are transmitted to the upper computer system through the RS485 communication serial port. The vehicle-mounted terminal serves as a display, capable of showing sensor data in real time and implementing automatic sound and light alarm functions, among others. The overall structure of the cotton picker fire detection system is shown in Figure 1. The data collected by the CO sensors and infrared sensors, which can detect the corresponding gas concentration and temperature, will be analyzed using the fusion algorithm model to predict the likelihood of a fire. Neural networks can utilize the relevant information more rapidly and accurately, thereby enabling the cotton picker to perform advanced prediction of cotton fire information and fulfill the cotton picker fire warning function.

### 2.2. Hardware Design

**Figure 2:**
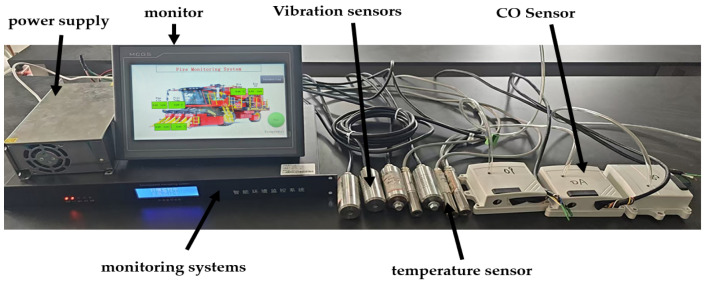
System hardware diagram.

The hardware components of the cotton picker fire detection system encompass infrared sensors, CO sensors, vibration sensors, displays, and an upper computer. Among them, temperature sensors and CO sensors are utilized to model relevant parameters, while vibration sensors collect data regarding the picking chamber of the cotton pickers and provide a reference for determining if the cotton picker is operating properly. The upper computer incorporates an embedded neural network algorithm, which is used to process the sensor data and assess the potential fire risk of the cotton picker. The hardware design system of the cotton picking machine is shown in Figure 2.

#### 2.2.1. In-Line Infrared Temperature Sensor Modules

**Figure 3:**
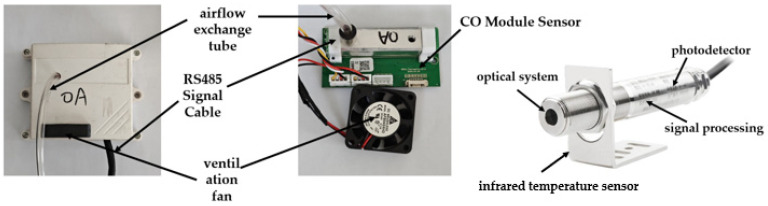
Temperature module and CO module diagram.

In order to guarantee the accuracy of temperature measurement, this study employs the infrared temperature sensor model FST600-400A (Firstrate Corporation, Changsha, Hunan Province, China) for non-contact temperature detection, as shown in Figure 3. This device utilizes thermal and photoelectric detectors to convert the received infrared radiation into electrical signals. Subsequently, the temperature value is calculated based on the fundamental law of radiation and presented through a display. This sensor exhibits a wide temperature measurement range, spanning from 0 °C to 1200 °C, and features an output signal of the RS485 four-wire system. It operates under a wide voltage range of 10 to 30 V, with a measurement accuracy of ±2% of the measured value. The spectral response range is 8 to 14 μm, and it is adapted to the ambient temperature of 0 to +60 °C, with a response time of 300 ms. Due to its non-contact measurements nature, it does not alter the temperature of the object under measurement. Additionally, it possesses a wide temperature measurement range, rapid response speed, and high sensitivity, rendering the measurement results authentic and reliable, thus meeting the requirements for detection temperature data in cotton picker fire situations.

#### 2.2.2. Infrared CO Module Sensor

In consideration of the special environment of cotton picker field operations, the JX-CO-103 CO sensor (China Shandong Weihai Jingxun Changtong Electronic Technology Co., Weihai, China) was selected for this study, as shown in Figure 3. The sensor operates based on the NDIR infrared absorption detection principle, which determines the CO concentration by measuring the intensity change in infrared light after passing through the gas to be measured. It integrates advanced optical circuits, precision circuits, and intelligent software to form a high-performance infrared CO sensor module. It offers a measuring range of 0~1,000,000 ppm, a resolution of 1 ppm, an accuracy of ±5% F.S. (2 °C), a response time of less than 30 s (2 °C), an operating voltage of 9~24 V, and is adapted to a humidity range of 0~95% RH (non-condensing dew), with a working temperature ranging from −10 °C to 75 °C and an infrared light source wavelength band of 2~14 μm. To ensure efficient and stable operation and effective detection of gas concentration in dusty and other impure environments, the sensor is equipped with a protection system to safeguard the sensor and ensure its efficient and stable operation. Moreover, the sensor is also designed with a protective housing, a ventilation fan, and an airflow exchange tube, as shown in the figure above. The signal transmission adopts RS485 communication mode, and the ventilation fan utilizes an independent power supply to ensure the stability of the working voltage.

#### 2.2.3. Upper Computer and Display Unit

The display module adopts the MGCS touch screen, formally referred to as Kunlun Tongshi touch screen, which is a type of high-performance human–machine interface (HMI) device engineered for industrial automation and process control applications. It comes pre-installed with MCGS embedded configuration software (McgsPro 3.3.6.6354), enabling it to support complex data display and processing requirements. Moreover, it offers a wealth of interface options, such as RS232, RS485 serial port, USB interface, Ethernet interface, etc., to satisfy diversified communication demands, and, at the same time, it has an IP65-level protection rating, allowing it to work stably in harsh environments, thus meeting the requirements of the cotton picker’s working environment.

**Figure 4:**
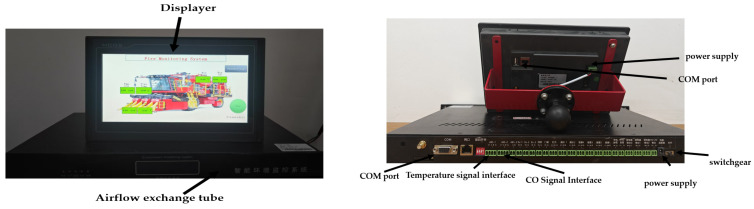
Software system diagram.

The cotton picker fire detection system is equipped with an ARM-core processor and its main frequency reaches up to 600 MHz or even higher, ensuring the efficient operation of the system. The back-end is integrated with a COM port, RS485 interface, power outage protection function, sound and light alarm function, etc., supporting serial multi-protocol communication; it can also directly communicate with the infrared temperature sensors and infrared CO module sensors, featuring superior performance and lower power consumption, as shown in Figure 4. When the sound and light alarm meets the fire detection and warning conditions of the cotton picker, it can display the fire alarm in real time.

### 2.3. Software Design

**Figure 5:**
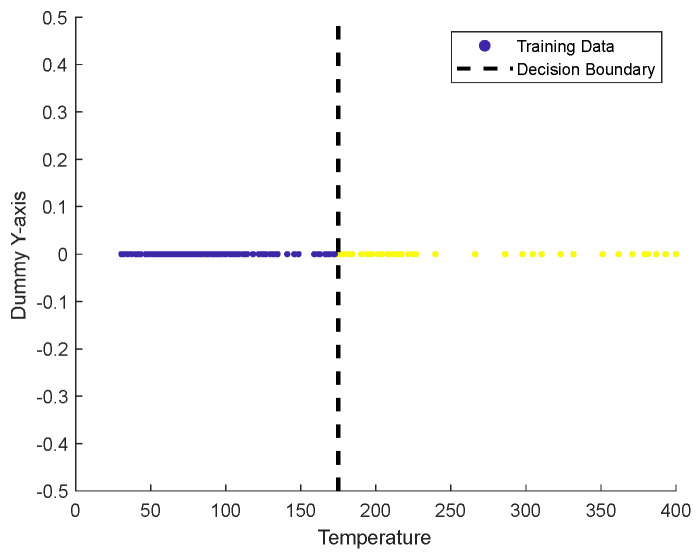
Threshold graph.

To implement the neural network for cotton picker fire warning, this study devised an optimization algorithm based on the BP neural network. By integrating the gray wolf and particle swarm algorithms, the optimal parameters of the neural network were determined. Subsequently, the optimized neural network was deployed to the upper computer to achieve the real-time data prediction function of the upper computer. The upper computer collects data via the infrared sensor and CO sensor, reads the data, and conducts analysis. By training the data multiple times using the SVM algorithm in MATLAB (2022b), the critical value of 170 °C between the burning and non-burning states of cotton was identified. The value obtained by the SVM algorithm is presented in Figure 5.

**Figure 6:**
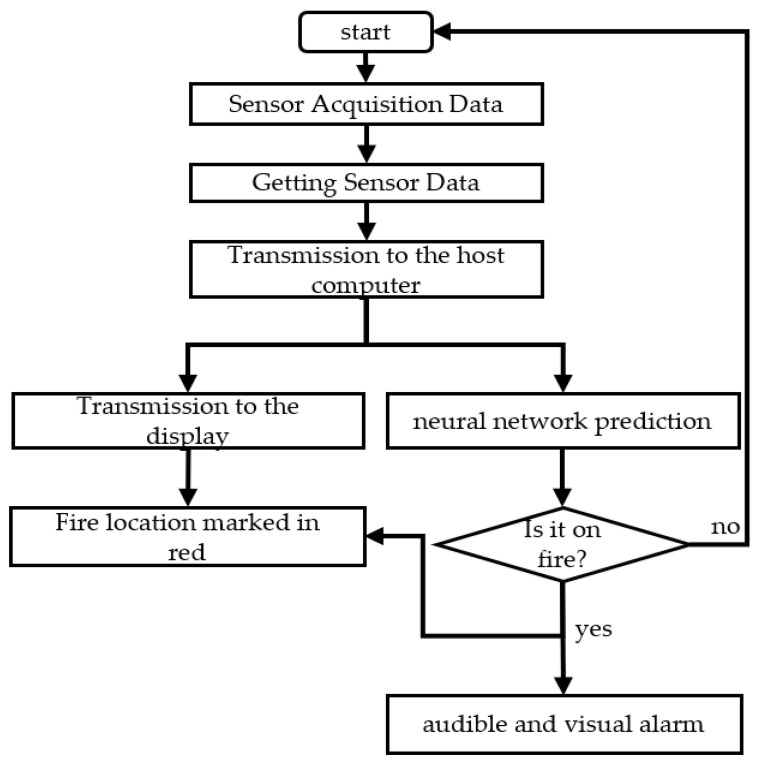
System flow chart.

Combined with the findings of Liang [16], who pointed out that the cotton combustion temperature is 150 °C, this study decided to set 160 °C as the initial alarm value. Regarding the CO concentration, concerning the low concentration in the normal atmosphere as referred to in reference [5], the initial alarm threshold is set to 100 PPM. This initial alarm threshold is established to prevent false alarms during system leakage. For the system operation, refer to Figure 6, where the operation flow of the system can be clearly observed.

#### 2.3.1. Access to Data

**Figure 7:**
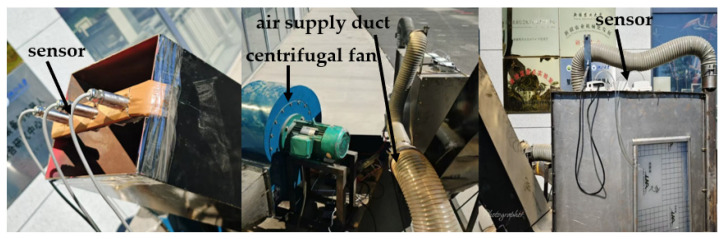
Data acquisition bench.

In order to explore the performance of the improved algorithm, a test bench was constructed according to the structure of the cotton picker. The data obtained are presented in Figure 7. From the figure of the test bench, the installation of the sensors and the data acquisition method can be seen. The data collected by the sensors are transmitted to the upper computer via RS485. The upper computer can then perform real-time data processing and storage, and the data will be exported for model training.

#### 2.3.2. Software Algorithm Design

**Figure 8:**
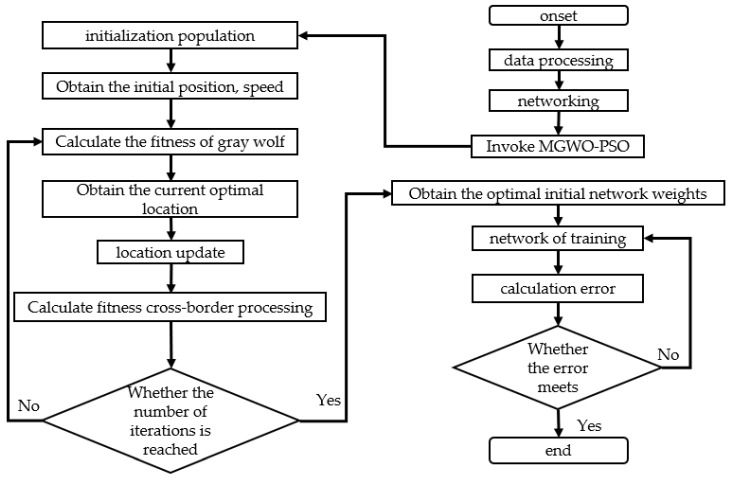
Flowchart of the improved GWO-PSO-BP algorithm.

The BP neural network possesses reverse learning capabilities. Through training error backpropagation, the error is propagated layer by layer from the output layer back to the input layer through the hidden layer The error is then apportioned to all units in each layer, thereby obtaining the error signal for each unit in each layer [17]. This network exhibits excellent nonlinear mapping capabilities, making it suitable for solving nonlinear problems. However, in fire prediction, it has drawbacks such as the vanishing gradient problem, a tendency to fall into the local optimum, a relatively long learning process, and a slow convergence speed [18]. To optimize the BP neural network, a fusion algorithm is employed: the particle swarm algorithm utilizes the memory and information-sharing characteristics of seed particles to search for the optimal solution [19], which accelerates the gray wolf algorithm in finding the optimal solution. This enables the gray wolf individuals to locate the optimal solution more rapidly. Meanwhile, the mutation characteristics of genetic variation are introduced. The mutation operator is an essential operation in the genetic algorithm for generating a new individual [20]. The mutation-induced characteristics enhance the population’s adaptability to the environment. It is a method in the field of engineering that adopts a mathematical model of biological evolutionary theory to solve for the optimal solution [21]. The core equations of the fusion algorithm are as follows:

(1) $$Y_{\mathrm{ij}}(t+1)=Y_{\mathrm{ij}}(t)+F\cdot ({\mathrm{ub}}_{j}-{\mathrm{lb}}_{j})\cdot (2r-1)$$

(2) $$v_{\mathrm{ij}}(t+1)=w\cdot (v_{\mathrm{ij}}(t)+c1\cdot r1\cdot (x_{1j}-x_{\mathrm{ij}})+c2\cdot r2\cdot (x_{2j}-x_{\mathrm{ij}})+c3\cdot r3\cdot (x_{3j}-x_{\mathrm{ij}}))$$

(3) $$x_{\mathrm{ij}}(t+1)=x_{\mathrm{ij}}(t)+v_{\mathrm{ij}}(t+1)\;$$

where Y_ij_ is the current position of the gray wolf, F is the variation factor used to control the degree of variation, and r is a random number between 0 and 1. v_ij_ is the velocity of particle i in the jth dimension, v(t + 1) is the position at the next moment, and c1 and c2 are the learning factors corresponding to the particle’s own optimal position (personal best, pbest) and the social optimal position (global best, gbest), respectively, indicating the extent of their influence. The flowchart of the optimized neural network is shown in Figure 8.

**Figure 9:**
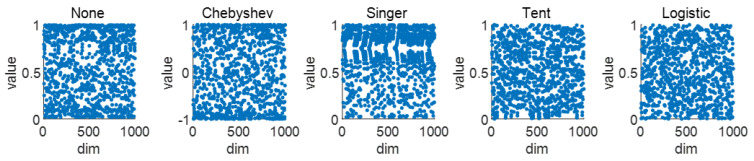
Population spatial comparison.

For gray wolf’s random search for prey, the distribution of the population also has a certain influence on the speed of the prey search. A chaotic search strategy is adopted to generate the initial population, ensuring the distribution of the initial population in the search space [22]. A more uniform distribution space plays an optimized role in the algorithm optimization. Therefore, a simple logistic chaotic map is introduced to initialize the population, aiming to improve the algorithm’s performance. As shown in Figure 9, five population chaotic maps are compared: none mapping, Chebyshev mapping, Singer mapping, tent mapping, and logistic mapping. It is found that the logistic and tent mappings result in a more even distribution compared to other mapping populations. However, the logistic expression is simpler with relatively lower computational complexity. Thus, the logistic chaotic mapping is better. The equations of logistic and tent mapping are shown in Equations (4) and (5), where a is the control parameter and μ is the chaotic parameter.

Logistic map:

(4) $$y_{i+1}=a\cdot y_{i}\cdot (1-y_{i})$$

Tent mapping:

(5) $$x_{n+1}\left\{\begin{array}{l} \mu \cdot x_{n}\\ \mu \cdot (1-x_{n}) \end{array}\right.$$

#### 2.3.3. Fusion Algorithm for Mutation Operator Optimization

In order to assess the performance of the enhanced algorithm model, several evaluation metrics are employed, including the root mean square error (RMSE), mean absolute error (MAE), area under the curve (Auc), and Pearson’s coefficient R. RMSE is capable of reflecting the accuracy of the model’s prediction outcomes. Pearson’s coefficient can indicate the model’s relevance, with the value of R approaching 1 signifying a more accurate model and stronger generalization ability [23]. The area enclosed by the ROC curve is defined as Auc, and a larger Auc value implies a more robust model. The equations for RMSE and Pearson’s correlation coefficient are as follows:

(6) $$\mathrm{RMSE}=\sqrt{1/n\sum_{i=1}^{n}(y_{i}-\overset{\wedge }{y_{i}}{)}^{2}}$$

(7) $$R=\frac{Cov(y_{i},y_{i})}{\sqrt{Var(y_{i})\cdot Var(y_{i})}}$$

y_i_: actual value, $\overset{\wedge }{y_{i}}$: projected value, n: sample size.

By training the model, the table is obtained as shown in Table 1.

**Figure 10:**
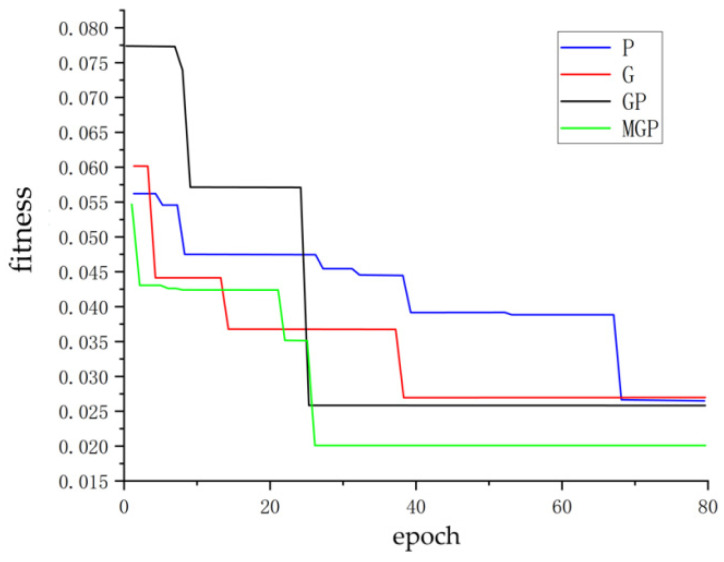
Iteration comparison chart.

Based on the data presented in the table, a detailed analysis of the performance of different algorithms with respect to the four evaluation indices can be carried out. The MGWO-PSO algorithm exhibits superior performance in terms of RMSE, which implies that the difference between the predicted values of this algorithm is the smallest, thus resulting in the highest prediction accuracy. Simultaneously, the MGWO-PSO algorithm attains a relatively favorable value for AUC, which indicates a better classification performance. However, during simulation, it is observed that the area under the Roc curve of the improved model is slightly smaller than that of the original model. This phenomenon can be attributed to the introduction of the variance operator. Although the variance operator boosts the search capability of the gray wolf, the influence of the variance and its intensity leads to a slightly lower robustness of the model compared to the original algorithm. Nevertheless, when considering all aspects comprehensively, since the difference in Auc is not substantial, in comparison, the MGWO-PSO algorithm achieves the highest value of 0.91929 in terms of R (Pearson’s correlation coefficient), which indicates that there exists the strongest linear relationship between its predicted value and the true value, thereby enabling it to perform excellently in fire prediction and classification tasks. For the comparison of the four optimized algorithms, the population size is set to 30, the maximum number of iterations is set to 80, and the number of neutral network training times is set to 500. As depicted in Figure 10, it can be observed that the improved algorithm can complete its iterations earlier and the iteration value is smaller. This suggests that the quality of the solution obtained by the improved algorithm is better, which is beneficial for the accomplishment of the fire task. (P: PSO algorithm, G: GWO algorithm, GP: PSO-GWO algorithm, MGP: MGWO-PSO algorithm).

### 2.4. Experimental Validation of Fire Conditions Based on Optimization Algorithms

**Figure 11:**
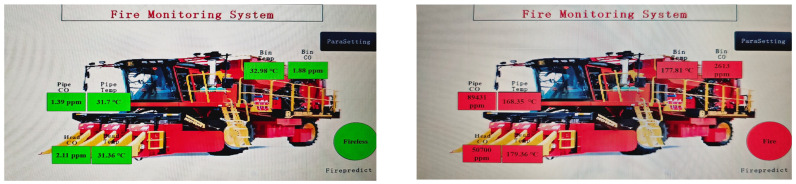
Display of real-time forecast data graphs.

In order to verify the actual predictive performance of the algorithms, they are installed onto the upper computer and tested through the test bed. Data acquisition is achieved through the ignition of cotton, with sensors collecting data. The RS485 serial bus facilitates the connection between the computer and the control system for experimental testing, and the collected data are shared on the display, as depicted in Figure 11, and the obtained data are presented in Table 2. According to the data collected from the actual test bed, no data packet loss phenomenon was observed, and thus the table does not include the leakage rate data.

As indicated by the tabular data, significant differences in the performance among different algorithms can be observed in terms of accuracy and false alarm rates. Specifically, the BP algorithm exhibits an accuracy rate of 82.86% and a false alarm rate of 17.14%. The PSO algorithm shows an accuracy of 91.43% and a false alarm rate of 8.57%. The GWO algorithm further boosts the accuracy rate to 92.38% while reducing the false alarm rate to 7.62%. The GWO-PSO algorithm attains an accuracy rate of 94.29% and a false alarm rate of 5.71%. Notably, the MGWO-PSO algorithm performs optimally, with an accuracy rate of 96.10% and a false alarm rate of merely 3.9%. This indicates that the MGWO-PSO algorithm not only has the highest accuracy rate but also the lowest false alarm rate, thereby demonstrating its substantial advantage in task completion effectiveness. It can be observed that in the prediction and identification of the cotton picker fire task, the improved algorithm is more effective compared to other algorithms and is capable of better fulfilling the fire detection requirements.

### 2.5. Field Tests

**Figure 12:**
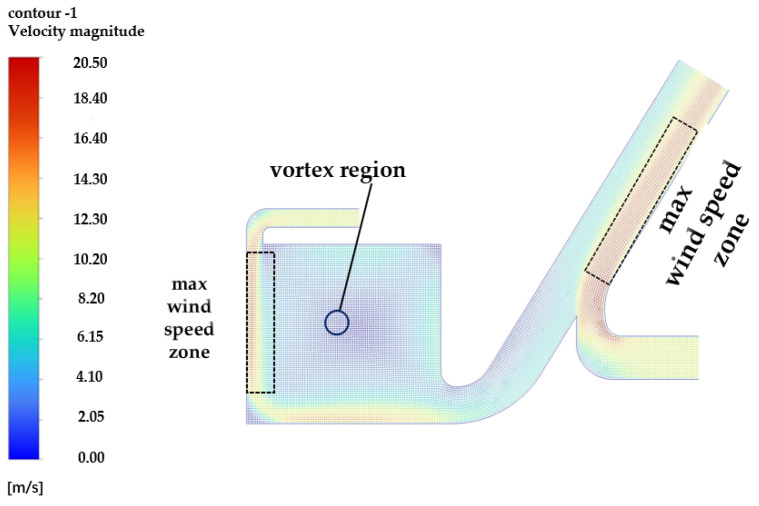
Simulation of the wind field inside the picking room.

**Figure 13:**
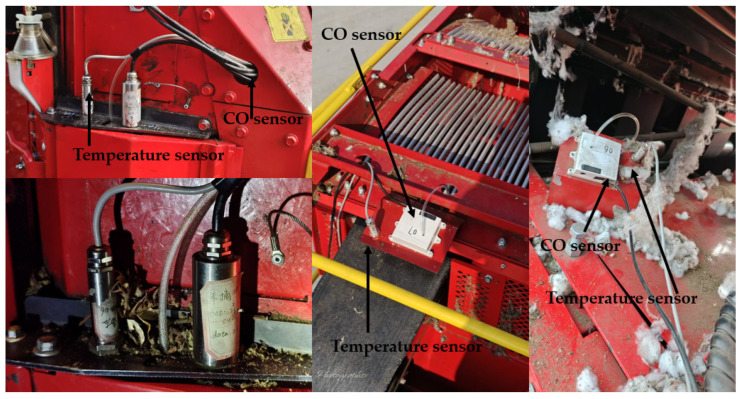
Cotton picker operation sensor location map.

(1) The trained prediction model is employed for the cotton picker’ fire task and can be installed in the bit machine for real-time measurement. To enhance the accuracy of data measurement, an in-depth discussion on the sensor installation position is necessary. In this study, the picking chamber and the cotton transport channel are selected as the research objects, and the corresponding simulation model is established, as shown in Figure 12. Due to the presence of a wind field in the picking chamber, the standard k-ε (k-epsilon) model is adopted for model selection, which is widely recognized for its relatively simple form and good robustness. During the simulation process, the inlet wind speed is set at 25 m/s based on the actual wind speed of the cotton picker fan during operation. Based on the simulated velocity field distribution results shown in Figure 2, it can be observed that there are several vortex zones and maximum wind speed zones within the channel. On one hand, the vortex zone may lead to smoke dilution or uneven mixing due to the unstable airflow. On the other hand, in the region of maximum wind speed, the excessively fast airflow might cause smoke particles to pass through the sensor sampling zone rapidly, resulting in detection delay or omission. Considering these factors and in combination with the results of the simulated velocity field analysis, it is recommended that the smoke sensor be installed at a more suitable location, such as the bottom of the picking head or the upper part of the cotton tube. This ensures a relatively stable airflow environment and facilitates effective smoke detection. As for the location of the sensor in the cotton box, it is advisable to place the sensor at the top of the cotton box for better data collection.

(2) After determining the position of the picking chamber sensor, upon powering on the cotton picker, the sensors start to operate following initialization, collecting data and transmitting it in real time to the upper computer. The upper computer, upon receiving the data, transmits it to the display to show the real-time temperature. Simultaneously, real-time data prediction, judgment of the data, and output of the prediction results are performed on the display. The actual position of the sensor on the working cotton picker is shown in Figure 13.

During the operation of the cotton picker, data predictions for three time periods are based on the data presented in Table 3. The average accuracy of the data obtained from the three real-time tests is 96.06%, and the false alarm rate is below 5%, indicating that the modified program is viable.

## 3. Conclusions

The following conclusions can be drawn by integrating the algorithm evaluation tests conducted in the laboratory using the test bed and actual tests performed on the cotton picker in the field:

(1) The test bed is constructed by referring to the structure of the cotton picker, aiming to replicate the picking chamber part and other components of the cotton picker as closely as possible. By using a centrifugal fan to simulate the air supply system of the cotton picker, data are collected from the sensors, and the model training is carried out on the upper computer, with the objective of selecting the algorithmic model that is more proficient in handling cotton picker fire situations.
(2) Through the fusion algorithm and the introduction of mutation operations, rapid optimization is achieved. Based on the simulation analysis, the improved algorithm attains superior performance compared to other algorithms in terms of various indices, enabling the BP neural network to perform fire prediction tasks more efficiently.
(3) In the actual operation of the cotton picker, experimental data collected using the SVM algorithm, along with the critical value of cotton combustion predicted by the relevant literature as the initial reference threshold, are utilized by the upper computer to predict fire condition and share the information on the display. It can be concluded that the model’s performance in the actual fire detection of the cotton pickers meets expectations.
(4) This study primarily focuses on two critical factors related to cotton burning. However, for future work, improving the data collection by affected smoke sensors and other sensors, as well as exploring the utilization of more effective sensors for fire detection, remains a crucial area for enhancement.

## Figures and Tables

**Table 1:** Evaluation indices without algorithms.

Algorithm	RMSE	R	AUC	MAE
bp	0.32049	0.83754	0.79979	0.24231
PSO	0.20578	0.84904	0.81668	0.22971
GWO	0.19035	0.89759	0.85654	0.22238
GWO-PSO	0.16664	0.91381	0.99192	0.21336
MGWO-PSO	0.09928	0.96929	0.99038	0.17077

**Table 2:** Experimental test data of the algorithm.

Algorithms	Bp	PSO	GWO	GWO-PSO	MGWO-PSO
accuracy	82.86%	91.43%	92.38%	94.29%	96.10%
false positive	17.14%	8.57%	7.62%	5.71%	3.9%

**Table 3:** Data table of field experiment.

Groups	Number of Times Data	Accurate Count	Accuracy	False Positive
Group 1	500	477	95.4	4.6%
Group 2	500	481	96.2%	3.8%
Group 3	500	483	96.6%	3.4%

## Data Availability

The dataset used in this research is available upon valid request to any of the authors of this research article.
